# Notes on Epidemic and Other Diseases Which Have Prevailed in the United States, Since Their First Settlement

**Published:** 1850-09

**Authors:** C. A. Lee


ART. III.—*Notes on Epidemic and other diseases which have prevailed in the United States, since their first settlement.*—By C. A. Lee, M. D.

(*Continued from page* 974, vol. v.)

In the winter and spring of 1761, a severe influenza prevailed over the Northern and Middle States, and the West India Islands. It began with a severe pain in the head and limbs, a sensation of coldness, shiverings succeeded by great heat, running at the nose, troublesome cough, and bilious symptoms. In May it seemed to run into a malignant fever; many aged people fell victims to it; the lungs becoming loaded with a thick, viscid phlegm, attended with cough, great prostration, laborious respiration, pains in the chest, præcordia and limbs, under which they speedily succumbed. (Webster.) In the summer of the same year, an infectious bilious fever prevailed at Charleston, S. C. The summer of 1762, was extremely hot and dry, scarcely any rain having fallen from June to September; the bilious fever proved very malignant and fatal in Philadelphia.

The summer of 1763 was wet and unhealthy. The Indians on the Nantucket, were attacked with "bilious fever," in August, and between that time and February following, their number was reduced from 358 to 136. Thirty-six only recovered out of 258, who were attacked. The disease is said to have "commenced with high fever, and ended in typhus, in about five days." It appeared to be infectious only among the Indians; for no whites were attacked, although they associated freely with the diseased. Persons of a mixed blood were attacked but recovered. None died, except of full Indian blood. The Indians on Martha's Vineyard were attacked with the same disease in December, of the same year, and out of 52 patients, 39 died. (Phil. Trans. and Lond. Mag., 1764. Hist. Coll, vol. 3, 158.) Severe anginas prevailed during several of the following years, in various parts of the United States; in 1772, epidemic catarrh was quite rife, and was followed by the measles, which proved very fatal, 90 children having died of it in Charleston, S. C. A typhoid fever swept off some 50 or more inhabitants of Cape Cod. The measles was succeeded by fatal anginas, and an epidemic croup which carried off many children, in some places, scarcely a single person recovered, who was attacked. The dysentery was very prevalent in 1773, in New Haven and East Haven, Connecticut, and Salem, Massachusetts, and put on a peculiarly malignant type. Cynanche maligna, would seem to have been very general over a considerable part of the United States, and very fatal, also, for several years, about this period; while croup, measles, scarlet fever, and dysentery, were also extremely prevalent and destructive.

During the year 1776, the dysentery prevailed over every part of the country, and very fatal to the American troops at New York, and Ticonderoga—Of 13,000 men, one half only were fit for duty. It was a general opinion that the disease spread by infection, and was carried by the soldiers returning to their homes. It had, however, proved very fatal in former years, as from 1749 to 1753; and in 1775, 100 of the inhabitants of Danbury, Connecticut, were swept off by this disease, in a population of about 1000. Typhus fevers were rife in 1778, and an "infectious bilious fever" prevailed in Philadelphia, during the summer and autumn, after the British left the city. The winter of 1780 was one of the coldest ever known in the United States. Webster says, that "not only all the rivers, but the harbors and bays in the United States as far South as Virginia, were fast bound with ice. Loaded sleds passed from Staten Island to New York; the Sound between Long Island and the main land was frozen into a solid highway, where it is several miles in breadth. Chesapeake Bay at Annapolis, where the breadth is 5½ miles, sustained, also, loaded carriages." —(On Pestilence, vol. 1. p. 266.) Dr. Rush states, (vol. I, 123,) that catarrhal complaints were very common among children during the spring of 1781, and the bilious remittent fever was epidemic in Philadelphia, during the summer and autumn, accompanied by such acute pains in the back, hips, and neck, as to obtain the name of the "*break-bone fever.*" The influenza, also, swept over the country during the spring of the same year; beginning with a severe pain in the head, prostration of strength, coldness and chills, &c. The cough was troublesome. The disease usually terminated about the thirteenth or fourteenth day—although sometimes on the eighth or ninth. The same disease pervaded the eastern hemisphere the year following. The scarlatina appeared in Philadelphia in August, 1783, and in September became epidemic, and extended over New England, in 1784, where it continued to prevail with more or less violence for five or six years continuously. The measles and cynancha maligna were also, very prevalent during the same period. The Dysentery and the usual autumnal fevers were not, however, as general or fatal, as they had been in previous years. Canine madness began to rage and spread in 1785, in all parts of the Northern States, and many of the inferior animals became infected,—especially foxes, wolves, and horned cattle. The newspapers in every part of the country abounded in cases of hydrophobia, while at the same time, malignant scarlatina was extremely rife and fatal. About the same period the ravages of the wheat insect, (weevil?) erroneously called the "Hessian Fly," were very destructive. It seems to have been introduced into the United States about the year 1776; first appearing on Long Island; then spreading over New Jersey, traveling at the rate of about 20 miles annually; till in 1785, it became so destructive to the grain, that the English Government issued a proclamation, dated June 25th, 1788, prohibiting the importation of American wheat. The Agricultural Society of Pennsylvania, however, in a report published at the time, (Museum, vol. 4. 244,) express the opinion that it is "the *plant* alone, which is injured by the insect; that the grain is sound and good, and that the insect is not propagated by sowing wheat, which grew on fields infected by it." The scarlet fever and hydrophobia continued their ravages throughout New England and the Middle States during the year 1786; the former, after sweeping away whole families of children.

Noah Webster describes a remarkable halo that appeared in Connecticut in May, 1789, and states, that a clergyman wrote an essay on the occasion, full of gloomy predictions, regarding the halo as certain evidence that "the arm of the Lord was extended in wrath over our land."

In the spring of 1789, there was something like a famine in the Northern States. In Vermont, the people found a new kind of food "in tad-poles, boiled with pea-straw." "Four potatoes sold for nine-pence." The crops, especially corn, had failed, to a great extent, during the preceding year, and "the cod fish were sickly." Scurvy and fluxes were consequently very rife, and cattle perished in large numbers. Hydrophobia again raged, and in Maryland there was great mortality among horses.

In the fall of 1788, the bills of mortality for New York and Philadelphia show that the measles prevailed extensively, and we find that this disease became epidemic over the Northern States the same year. The influenza, or epidemic catarrh, also, became epidemic, though the precise time and place of its appearance are not ascertained. We first hear of it in New York and Philadelphia, in September, 1789. Dr. Rush thought it was brought to Philadelphia by the members of Congress, who returned from New York in October. It soon spread over the whole country, appearing in Connecticut about the middle of October. It was generally regarded as highly infectious. It invaded the wilderness, attacked the Indians, spread over the ocean, seized the mariner a hundred leagues from land, invaded the West Indies about the same time it did the Northern States; in short, it overspread America, from the 15th to the 45th degree of latitude, in six or eight weeks, proving, as usual, very fatal to the aged, and those of debilitated habits and broken constitutions. It seemed in some places, to replace scarlatina; in others, to alternate with it; in some, it raged coincidentally. One marked feature of both these diseases, at this period, was the bilious character they assumed, which continued to mark the prevailing complaints for several subsequent years. Noah Webster informs us, (On Pestilence, vol. I, p. 290,) that the scarlet fever did not spread over the country in 1790. "It was little known," he remarks, "in the Northern States, till two years after—this is among the proofs that this disease does not depend on infection for its propagation. If infection was its only, or principal means of propagation, the fomites existed in great abundance, in particular places, in 1790, and sufficient to have spread it over the United States. But a disease, however infectious, will not spread far in an atmosphere that will not generate it. Indeed, scarcely a year passes, in which sporadic cases of scarlatina, or anginas, of other kinds, do not appear in particular places, *but they never spread without some uncommon concurrence of causes.*" This latter, is a very true remark. What these causes or circumstances are, we are, as yet, to a great extent, ignorant; but that they exist, no one can doubt. To them, in connection with a pre-existing *virus*, or *contagious principle*, we must always look for a true explanation of the prevalence of epidemic diseases.

A second epidemic influenza raged in the Spring of 1790, pervading, as before, all parts of the country, but more violent than that of the preceding year, sweeping off large numbers of consumptive persons, and hard drinkers. Mr. N. Webster notices as a remarkable fact, that 14,000 shad were caught in a single draught, by some fishermen, at the Narrows, near New York, in April, of this year, viewing it as a sure precursor of pestilence, along with meteors, volcanic eruptions, hurricanes, severe cold and heat, earthquakes, aurora borealis, &c., &c. "It will be noticed," he remarks, "that the pestilential fever, which has prevailed for many years past, first appeared in New York, in the autumn succeeding this singular draft of fish." (Læ cit.) "It may," he adds in a note, "excite surprise that there should be supposed a connection between an uncommon abundance of fish and pestilence. But the theory that resolves this into the unusual power of excitement is rational. The state of the elements that causes pestilence, always produces unusual numbers of insects; and often the human race is more prolific than at other times." During the autumn of 1791, bilious remittents were uncommonly prevalent in Philadelphia, according to Dr. Rush, assuming an inflammatory form, requiring repeated bleedings. The yellow fever, also, carried off more than 200 persons, along the banks of the East River in New York. In 1792, the same disease ravaged Charleston, S. C. Scarlatina became epidemic, also, in New England and the Middle States, and swept off multitudes of children. The influenza prevailed extensively, also, the same year. The scarlet fever, at that period, is described as coming on with "slight influenza, stinging pains in the jaws and limbs, soreness in the muscles of the neck, with a slight fever." In addition to the above, a very fatal form of dysentery prevailed in some of the Northern and Middle States, in the summer and autumn of 1793, which in Coventry, Conn., is said to have proved fatal in every case attacked. A severe "lung fever" also, prevailed at Weathersfield, and some other places. In August, 1793, commenced that very fatal yellow fever, so graphically described by Dr. Rush, which, in a few weeks, carried off more than 4,000 victims, being preceded by influenza, scarlatina, and mild bilious remittents. The weather, during its prevalence, was very hot, dry, and sultry. (See Rush's works.) The scarlet fever prevailed extensively in Connecticut, in 1794; in the spring of 1795, it broke out in Boston, and continued to prevail in Massachusetts and New Hampshire, in 1796. It seems to have been about four years in extending from New York to Maine, some 400 miles. The same rate of progress, though in an opposite direction, was noticed in 1735.

On the 10th of June, 1794, the "bilious plague," or yellow fever, made its appearance in New Haven, Conn. Its origin has given rise to much discussion and dispute. The general belief was that the seeds of it were imported in a sloop from the West Indies. This, however, is denied by Webster, who traces its causes—1st, to the sickly state of the oysters on the Connecticut coast for several months preceding; 2d, to the multitude of caterpillars, which overrun New Haven, in the summer of 1794; 3d, to damaged pickled codfish, which lay exposed to the sun during the recess of the tide; 4th, to the garbage of shad and decaying clams. "So noisome," says Webster, "was the air of the place for some time before the fever appeared, that the proprietor of the wharf desisted from his usual morning visits before breakfast. The putrefaction of flesh, from 30 years' observation, I can testify, will not always produce disease. But in a pestilential state of air, the dissolution of flesh is unusually rapid, and the acid evolved, peculiarly noxious. In such circumstances, putrescent substances of all kinds appear to be powerful auxiliary causes of disease. The condition of the elements accelerates putrefaction, and that putrefaction in turn increases the deleterious quality of the air."—(Loc. cit.) Have we any right to assume "a pestilential state of the air," independent of any local causes? We should like to be informed what this "condition of the elements" is, which is always pre-supposed to exist, in advance of epidemics!

In July, 1795, the yellow fever broke out in the city of New York, and raged extensively along the low streets on the East River, in the region then and now called "the Swamp," and in the narrow alleys. The higher grounds in the centre of the city, and the western side of the island, were healthy as usual, and the disease, when carried to the healthy portions of the city, did not spread. The citizens mostly remained in the city. The deaths reached 730; 500 of whom were foreigners, chiefly Scotch and Irish. Then, as now, the excessive mortality of New York was due to the immense influx of foreigners, not seasoned to our climate, and whose habits of life are not regulated by proper hygienic laws. Dr. Treat, the health officer of the port, was one of the earliest victims. The disease was evidently infectious, where local circumstances were propitious, the same as the cholera, typhus, dysentery, &c., are at the present time. A few cases of yellow fever occurred at a small village called Mill River, near Fairfield, Conn., the same season, which were traced to infection from New York.

It is hardly necessary to state that the bilious remittent and intermittent fevers prevailed extensively in our new settlements, both at the south and west, and annually decimated the population. New York State, which was then just being brought under cultivation, bore the reputation of a very unhealthy country. Every new settler, almost, became subject to an attack of some form of malarious disease, and in many parts of the State, during the autumnal months, there was great distress and suffering, there not being a sufficient number of well persons to attend upon the sick. Few of us at the present day reflect at what an immense expenditure of strength, and health, and life, the broad fields of New York have been brought to their present state of cultivation. We, the degenerate and more effeminate sons of a hardier race, are reaping the rewards of their toil and labor, little mindful at what a sacrifice they were purchased. At that period, the bronchocele was also a very common complaint in our State, especially in the regions adjacent to the lakes; to what causes it should be attributed, we are entirely undecided.

(*To be Continued.*)

